# Learning from the world's oldest continuous culture — the circle of leadership: a new way to support the healthcare workforce

**DOI:** 10.3389/frhs.2026.1888055

**Published:** 2026-08-19

**Authors:** Leila Karimi, Dein Vindigni, Karen Hallam, Doa El-Ansary, James Collett, Sophia Xenos, Paul Callaghan

**Affiliations:** 1 School of Health and Biomedical Sciences, RMIT University, Melbourne, VIC, Australia; 2 School of Medicine and Healthcare Management, Caucasus University, Tbilisi, Georgia

**Keywords:** Aboriginal and Torres Strait Islander health workforce, circular leadership, collective leadership, cultural safety, healthcare workforce retention, indigenous leadership, wellbeing

## Abstract

Healthcare systems globally face workforce shortages, burnout, and attrition, intensified by the COVID-19 pandemic and the loss of experienced clinicians across disciplines. These pressures are particularly acute within the Aboriginal and Torres Strait Islander health workforce, where culturally unsafe workplaces, limited support structures, and high workloads continue to affect recruitment and retention. This Perspective proposes an Aboriginal-inspired Circular Leadership Model as a culturally grounded and relational approach to strengthening workforce wellbeing, retention, and leadership capacity within healthcare settings. Drawing on Aboriginal ways of knowing, being, and doing, the model integrates principles of kinship, connection, collective responsibility, storytelling, reflection, and shared decision-making. In contrast to traditional hierarchical leadership paradigms, circular leadership positions collective wellbeing at the center of leadership practice and emphasizes peer support, multi-generational learning, culturally safe mentorship, and community connection. This is a conceptual Perspective rather than a systematic review: the paper draws on Indigenous leadership scholarship, collective leadership research, and the healthcare workforce literature to discuss how Aboriginal leadership principles can complement existing approaches. Practical applications, including structured mentorship circles, collective wellbeing practices, and circular decision-making protocols, are presented alongside implementation considerations. The anticipated benefits of the model for wellbeing, retention, and leadership capacity are presented as hypotheses to be tested through the pilot evaluation proposed here, rather than as demonstrated outcomes. The model is offered primarily as a strategy to support and retain the Aboriginal and Torres Strait Islander health workforce; its potential relevance to the broader healthcare workforce is discussed as a secondary and as yet untested possibility.

## 1 Introduction

The World Health Organization has identified a projected global shortfall of over 10 million healthcare workers by 2030 (1). The COVID-19 pandemic exacerbated these shortages, contributing to burnout, attrition, and a re-evaluation of life-work balance among many healthcare professionals — a phenomenon often described as “the great resignation” (2). The pandemic and ongoing post-COVID period also placed unprecedented demands on the remaining workforce, creating a “perfect storm” of higher attrition alongside escalating demand. Of particular note is the significant loss of senior, highly skilled nurses, which has exacerbated concerns about retention of new graduates as hospitals struggle to retain nursing staff (3, 4). New nurse graduates are particularly vulnerable during transition to professional practice, especially given the reduced availability of senior nurses to support them (5).

While the nursing literature provides the most extensively documented evidence of these dynamics, similar pressures are reported across the wider healthcare workforce, including allied health, medical, and Aboriginal and Torres Strait Islander Health Workers and Health Practitioners (6, 7). We therefore frame this Perspective around the healthcare workforce as a whole, drawing on the nursing evidence base as the most developed case while recognizing that the circular leadership approach we propose is intended to support healthcare workers across roles and disciplines.

Drawing on Aboriginal knowledges and wisdoms may inform the practice of “Leading Self,” the foundational capacity that underpins effective leadership of others (8). Aboriginal traditions of yarning, deep listening, and reflection on Country (9, 10) offer culturally grounded pathways through which healthcare workers can develop self-awareness, the reflective capacity to process clinical and emotional demands, and the personal resilience that supports sustained engagement and retention.

In addressing the current crisis, shortfalls in the Aboriginal health workforce must be made a priority. An Aboriginal and Torres Strait Islander health workforce delivers better outcomes for Aboriginal and Torres Strait Islander peoples (7). The 2021 Census shows that only 3.1% of Aboriginal and Torres Strait Islander people aged 15 and over were employed in health-related occupations, approximately 60% of the rate of non-Indigenous Australians (11). National strategies have highlighted the urgent need to expand this workforce. Workforce data indicate critical shortages in doctors, nurses, Aboriginal Health Workers, and Aboriginal Health Practitioners in services supporting Aboriginal and Torres Strait Islander communities, with a 20%–30% decrease in full-time equivalent clinical staff per 1,000 population members and an approximate 50% increase in unfilled positions since the COVID-19 pandemic (12). Staffing shortages could be partially addressed by encouraging more Indigenous Australians into health careers, but supporting existing Indigenous health professionals to remain long-term is equally important (13).

## 2 Supporting the Aboriginal healthcare workforce

Recruiting, supporting, and retaining Aboriginal employees requires a culturally safe and appropriate work environment with a genuine, embedded culture of respect and appreciation, led and modeled by management (14). Table 1 identifies enablers and barriers that managers need to be aware of when creating a supportive workplace environment for the Aboriginal health workforce. Good practice to support Aboriginal staff is also good practice for all staff: by targeting the enablers and addressing the barriers, leaders can create a better workplace for everyone.

**Table 1:** Enablers of and barriers to Aboriginal health workforce retention.

Enablers	Barriers
Positive work environment Supportive and culturally competent workplace Coworker support Peer mentorship Access to clinical and cultural supervision Professional development opportunities Career pathways Job security, adequate remuneration, and salary parity Making a difference Recognition of Indigenous health roles Clarity of employer, employee, and community expectations Cultural respect and safety in the workplace Flexible working arrangements	Racism Limited funding Heavy workloads and demands Lack of support from management Lack of professional development opportunities Proximity to community Lack of mentoring and supervision Feeling undervalued Lack of life/work boundaries

Adapted from Lai et al. (13).

A case study by Deroy and Schütze (15) with a regional Aboriginal Community Controlled Health Service identified six central themes supporting retention: social accountability; teamwork and collaboration; cultural safety; supervision; professional advancement; and recognition. Supporting the healthcare workforce thus involves several complex workplace themes, most of which are reliant on culturally informed, authentic leadership.

## 3 Current leadership paradigms in Australian healthcare

Australian healthcare draws on a small number of established leadership paradigms. Transformational leadership is widely promoted with a particularly developed evidence base in nursing. Transactional leadership remains common in clinical and operational management. Authentic and servant leadership have gained increasing attention more recently, emphasizing integrity, self-awareness, and serving the needs of staff and patients.

Each paradigm has contributed to thinking about engagement, wellbeing, and retention. They share, however, a fundamentally dyadic and hierarchical structure: leadership flows from a designated leader to staff, with comparatively little emphasis on collective decision-making, kinship, connection to Country, or Indigenous ways of knowing, being, and doing. This can create a work environment in which decision-making is stagnated, communication stifled, and rivalry accelerated. As Callaghan and Gordon (8, p. 170) observe, a culture of fear cripples innovation and creativity; risk-averse behavior creates a chain reaction of reduced productivity, poor workplace morale, loss of good staff, and ultimately forfeiture of competitive advantage. The circular leadership approach described below is not proposed as a replacement for these paradigms; rather, it complements them by adding a relational, communal, and culturally grounded foundation.

## 4 An Aboriginal-inspired circular leadership model

We propose the Circular Leadership Model as a complementary approach grounded in Aboriginal ways of knowing, being, and doing — leadership traditions that have sustained Indigenous communities for over 60,000 years. We acknowledge and respect the fundamental right of Aboriginal individuals and communities to self-determination, and the problems Aboriginal healthcare workers face as captured by Callaghan and Gordon (8): “The system continues to fail us because our leaders are making decisions for us, not with us, meaning they don't understand our story and so target symptoms rather than cause.” Circular leadership is presented here to support self-determination in the context of the Aboriginal and broader healthcare workforce, where its relevance is particularly acute. The model is offered first and foremost as a culturally grounded approach for consideration by the Aboriginal and Torres Strait Islander health workforce, the context in which the retention problem is best documented and from which the model's principles derive. Whether, and under what conditions, the model transfers to mainstream healthcare settings or to workforces beyond healthcare is an empirical question that this Perspective does not claim to answer; we return to this scope limitation in the Discussion. In developing the model, we drew substantially on Leading from the Dreaming (8), co-authored by one of the present authors (PC). This relationship is disclosed in the conflict of interest statement. PC's involvement reflects a deliberate choice to include Aboriginal knowledge holders as co-developers of the model rather than as external sources only; at the same time, we acknowledge that the model's theoretical foundation and this Perspective share an author, and we have therefore sought to triangulate the model's principles against independent Indigenous leadership scholarship (9, 10, 13) and the collective leadership evidence base (16).

### 4.1 Theoretical foundations

In Aboriginal cultures, leadership is inherently circular rather than hierarchical (8). Rather than power flowing from the top down, leadership is distributed throughout the community, with different individuals contributing knowledge and wisdom as situations require. Callaghan and Gordon (8) capture this in the 8Rs Model of Leadership: Responsibility, Relationships, Respect, Recognition, Reflection, Results, Resilience, and Renewal. Aboriginal wellbeing and effective leadership are founded on kinship and connection: connection to Country, to Community, to relationships, and to story. For new healthcare workers who often feel isolated during early career transition, this principle of connection may offer a powerful antidote. As Perry (17) notes, Aboriginal leadership insights represent “a gentle walk to a better view,” not rejecting Western practices but integrating ancient wisdom with contemporary approaches. A 2022 Cochrane systematic review found that collective leadership is likely to improve leadership practices and may improve team performance, with documented outcomes including improved staff engagement, satisfaction, and mutual respect (16). An important boundary to this evidence must be stated plainly: the Cochrane review concerns collective leadership interventions in general, not the Aboriginal-inspired model proposed here. It establishes the plausibility of distributed, collective approaches to leadership as a workforce strategy; it does not test the specific combination of mentorship circles, circular decision-making, and collective responsibility for wellbeing that we describe. The anticipated benefits of the Circular Leadership Model therefore remain hypotheses until the pilot evaluation outlined in Section 5.2 has been undertaken.

### 4.2 The model

**Figure 1:**
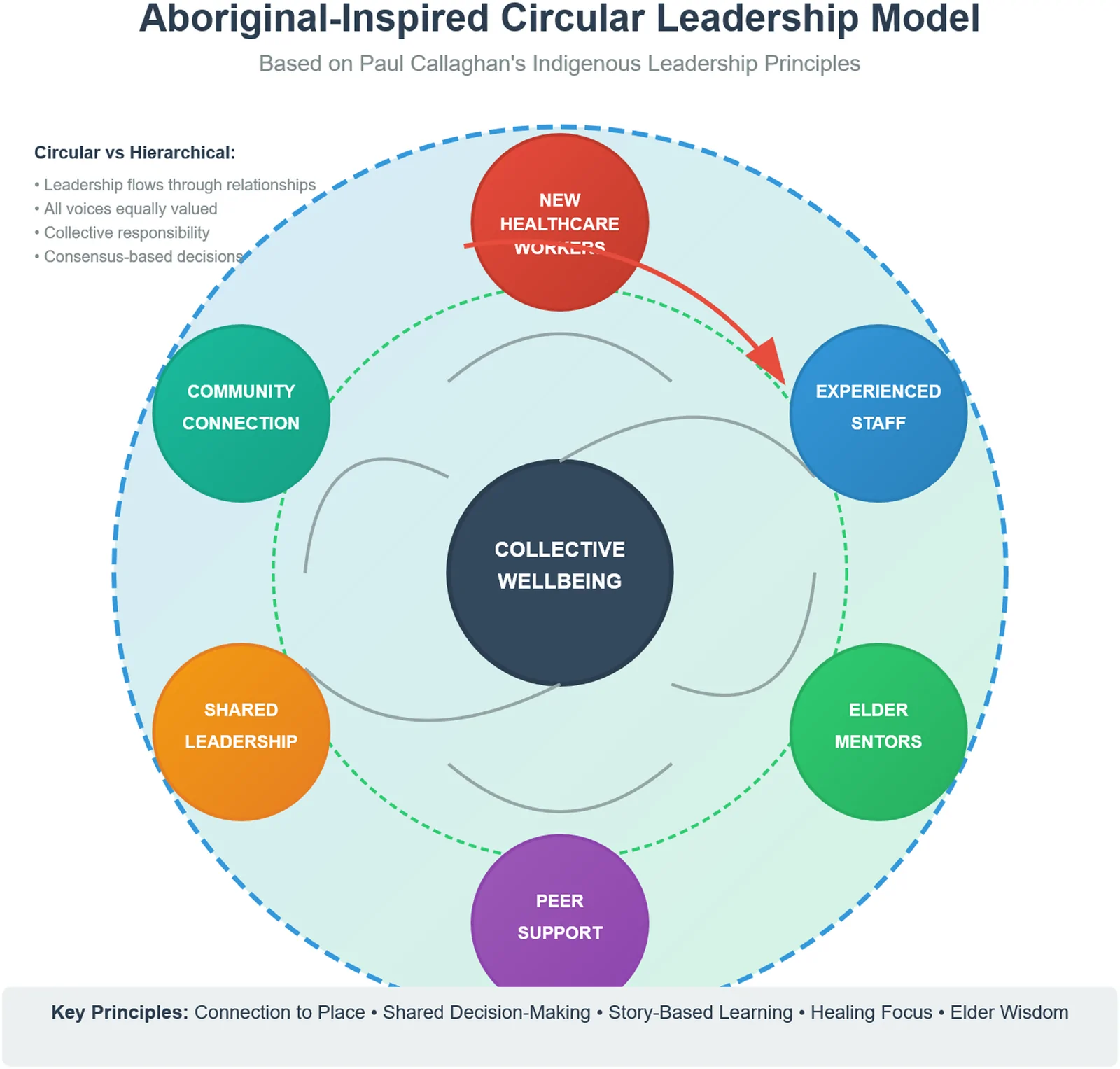
Aboriginal-inspired circular leadership model. Six stakeholder groups — new healthcare workers, experienced staff, Elder mentors, peer support, shared leadership, and community connection, are positioned around a central core of Collective Wellbeing. Connection lines demonstrate that leadership flows through relationships rather than hierarchy. Key principles include Connection to Place, Shared Decision-Making, Story-Based Learning, Healing Focus, and Elder Wisdom.

Figure 1 illustrates the Circular Leadership Model. Unlike traditional hierarchical structures, this model positions collective wellbeing at the center, with all participants — new healthcare workers, experienced staff, Elder mentors, peer support networks, shared leadership, and community connection — arranged in a circle of equal standing and responsibility, promoting authentic shared and two-way learning. In his book *Leading from the Dreaming*, Callaghan states: “In the Aboriginal way of thinking, wellbeing is only achieved if I am well, you are well, all people are well, Country is well, and all things in Country are well” (8, p. 183). This compels health professionals to acknowledge that focusing on the individual in isolation from the whole will not create sustained improvements in health outcomes. The model captures a key tenet of Aboriginal Lore that all things on this planet are connected: “Every creature and every thing comes from the Mother and has story … each of us is connected to all things. We are all one” (18), (p. 113).

### 4.3 Core components

The model has three interconnected components, illustrated in Supplementary Figure S1: structured mentorship circles, circular decision-making protocols, and collective responsibility for wellbeing.

#### 4.3.1 Structured mentorship circles

Drawing on Aboriginal communal learning and Elder wisdom-sharing, traditional one-on-one mentorship is reimagined through structured circles: peer support circles (new healthcare workers at similar career stages meeting regularly to share experiences and solutions); multi-generational learning circles (new graduates, experienced staff, and senior professionals learning as equals); cross-disciplinary connection (nurses, physicians, allied health and support staff collaborating); and place-based connection (regular opportunities to connect outside clinical settings).

#### 4.3.2 Circular decision-making

Aboriginal communities make decisions through circular processes in which all voices are heard and consensus is built collectively: circle meetings where each member has equal voice; consensus-building that prioritizes collective agreement over hierarchical decree; story-based learning that uses narrative and case examples to work through challenges; and structures that ensure new healthcare workers' perspectives are actively sought and valued.

In practice, circular decision-making does not require unanimity, and it does not rely on a formal vote. Following yarning traditions, the convening leader opens the circle by clearly stating the question to be considered and any non-negotiable institutional or regulatory constraints, for example, legislation, accreditation standards, budget envelope, and clinical governance requirements, within which the circle will operate. Each member then has the opportunity to speak without interruption. The convening leader synthesizes the discussion, names the emerging consensus, and tests that consensus with the group before recording the decision. Where clear consensus does not emerge within reasonable time, the convening leader retains the authority, and the accountability, to make the final decision, informed by the views expressed in the circle. The mechanism is therefore consensus-seeking with reserved authority rather than majority voting; this preserves operational decisiveness while ensuring that every voice has been genuinely heard.

#### 4.3.3 Collective responsibility for wellbeing

In Aboriginal cultures, individual wellbeing is inseparable from community wellbeing. Callaghan and Gordon (19) emphasize that Aboriginal healing focuses on “individuals, communities, and the Earth our Mother” as an interconnected whole. In the workplace this translates to shared workload models that distribute burden, collective check-ins focused on wellbeing, Elder support systems in which experienced staff proactively offer support rather than waiting for new workers to request help, and community healing practices that integrate connection to nature, gratitude, and collective reflection on meaning and purpose.

### 4.4 Examples in practice

While the integrated Circular Leadership Model is presented here as a conceptual framework awaiting formal pilot evaluation, its individual components are not new and resonate with documented Indigenous-led practices already in use across Australian healthcare and health professional education. Yarning circles are an established practice in nursing and midwifery education and in mental-health debriefing (10), where they support reflective learning and reduce professional isolation. Cultural mentor programs operating in a number of Australian health services pair early-career staff with senior cultural mentors, enacting the multi-generational learning principle described in Section 4.3.1. Indigenous Cadetship and traineeship programs combine paid employment, formal study, and structured peer and Elder mentoring, exemplifying the integrated mentorship-circle approach. These existing initiatives are not themselves the model we propose, but each demonstrates that its components are practicable. Pilots of the integrated model could productively connect, rather than replace, these existing structures.

Illustrative vignette. A regional hospital establishes a fortnightly peer support circle for first-year graduate healthcare workers, for example, nurses or allied health staff, complemented by a monthly multi-generational circle that brings together graduates, senior clinicians, an Aboriginal Health Practitioner, and (where culturally appropriate) a respected Elder. Conversations move between clinical challenges, cultural learning, and personal wellbeing, so early-career staff feel less professionally and personally isolated. The convening leader uses the daily handover as a brief check-in circle, and a monthly story-based round, each member sharing one shift story before discussion, supports consensus-seeking on operational decisions such as roster patterns or workflow change.

### 4.5 Anticipating concerns: accountability and performance

A reasonable critique of circular leadership is that distributing voice and decision-making risks blurring accountability and slowing the resolution of disagreements. Aboriginal leadership requires a leader to understand their responsibilities, obligations, and accountabilities to themselves, their team, their organization, and the external world (8, p. 35). We argue circular leadership does not abolish role-based accountability or formal management responsibilities. The accountable manager remains accountable. What changes is how decisions are reached, how dissent is heard, and how staff who are struggling are identified and supported. Disagreements tend to surface earlier rather than later, because dissent is built into circle protocols rather than suppressed by hierarchy. Performance management continues to follow standard processes; however, the supportive structures of the circle — early identification, peer support, and culturally safe conversations — may reduce the number of cases that escalate to formal procedures.

On the question of balancing collective input with institutional priorities, the convening manager opens each circle with a clear statement of any non-negotiable constraints (legislation, accreditation, budget, clinical governance). The circle then operates within that defined decision space. The accountable manager retains formal responsibility for the decision, including in circumstances where the manager personally disagrees with the group's preference and exercises reserved authority to decide otherwise. In such cases, transparency about the reasoning is itself a circle practice: the manager explains the decision to the circle, acknowledges the views expressed, and remains accountable for outcomes.

Where dissent persists after a decision has been made, three pathways are available and exist alongside the circle, not within it. First, the dissenting team member may request a follow-up yarning circle, facilitated by a culturally appropriate third party such as an Aboriginal Health Practitioner, an Elder mentor, or a workplace cultural advisor; this is a relational mediation step rather than a formal grievance step. Second, the matter may be escalated through standard organizational dispute, grievance, or industrial pathways, which continue to apply unchanged. Third, where the dissent concerns clinical risk or patient safety, existing professional incident-reporting and clinical-governance channels remain primary and override the circle. The role of the circle is to surface dissent constructively and early, not to displace these formal mechanisms.

## 5 Discussion

This Perspective has presented an Aboriginal-inspired Circular Leadership Model as a complementary approach to healthcare workforce attrition. The model draws on circular, kinship-based leadership traditions sustained over 60,000 years and integrates them with contemporary evidence on collective leadership (16). As noted in Section 4.1, that evidence supports collective leadership broadly rather than this specific model; the model's own effects on retention, wellbeing, and leadership capacity are propositions to be tested through the pilot evaluation described below, not findings reported here.

### 5.1 Adapting across leadership styles

Circular leadership is unlikely to feel equally natural to every leader. For leaders whose practice is more autocratic or strongly transactional, adoption will require a deliberate shift toward shared voice, slower consensus-building, and visible cultural humility. For leaders whose practice already aligns with transformational, authentic, or servant leadership, circular leadership is more likely to feel like an extension than a departure. A staged approach — introducing circle-based meetings and peer support first, before any larger shift in formal decision-making — is likely most effective. Coaching and reflective supervision for managers moving away from a directive style will be important.

### 5.2 Implementation and evaluation

We propose the model be piloted in a small number of healthcare settings, with co-design involving Aboriginal Elders and cultural advisors, clinical managers, and frontline staff. Evaluation should be mixed-methods and longitudinal. Suggested indicators include staff retention and turnover, intention-to-stay, psychological and cultural safety measures, participation in peer-support and multi-generational circles, and qualitative accounts of “Leading Self” and team functioning. Baseline measurement with follow-up at twelve and twenty-four months would allow change to be assessed.

Both the methods and the measures used to evaluate this work need to be culturally appropriate. Qualitative components should privilege yarning-based interviews and group yarning circles (9, 10), which themselves enact the relational principles of the model. The Most Significant Change technique offers a complementary mechanism by which staff and community members can identify the changes they consider most meaningful, capturing outcomes that standardized scales may miss. Quantitative components may include validated cultural safety self-assessment tools (for example, organizational cultural safety scales used in Aboriginal and Torres Strait Islander health settings), psychological safety scales, retention and intention-to-stay measures, and tracking of participation in circle activities. Evaluation should also center Aboriginal and Torres Strait Islander voice in interpretation: data ownership, sense-making, and reporting should follow Indigenous Data Sovereignty principles, with analysis sessions co-conducted with community and cultural advisors rather than solely by external research teams. Where the pilot site has an existing Aboriginal and Torres Strait Islander Health Worker, Aboriginal Health Practitioner, or cultural governance group, that group should hold a formal role in the evaluation governance from the outset, including sign-off on findings before publication.

### 5.3 Cost and benefit

Managers will reasonably ask what circular leadership costs. The additional protected time required is modest: a fortnightly peer support circle and a monthly multi-generational circle add a small number of hours per staff member per month. A short daily check-in circle can replace, rather than be added to, existing handover practice. Some of these hours will displace less effective existing meetings; others will be genuinely additional and require funding or roster adjustment.

These costs need to be weighed against the cost of inaction. Current attrition trajectories and reduced working hours are, on existing evidence, unsustainable (2, 20). The cost of healthcare worker turnover — most extensively documented in the nursing literature, including recruitment, orientation, productivity loss, and loss of organizational and clinical knowledge — has been documented internationally (21). Should the proposed pilot evaluation bear out the model's anticipated effects, even modest improvements in retention could plausibly offset the marginal short-term cost of additional protected time. Equally important, but harder to express financially, is the conservation of clinical and cultural knowledge that occurs when senior staff have structured time to nurture early-career colleagues.

## 6 Conclusion

Circular leadership represents a culturally grounded and implementable proposition in response to one of healthcare's most pressing workforce challenges. Its individual components are supported by existing evidence and documented practice; the effects of the integrated model on retention, wellbeing, and leadership capacity are hypotheses awaiting pilot evaluation. The model is offered as a complement to existing leadership paradigms, developed for and with the Aboriginal and Torres Strait Islander health workforce. Any extension to mainstream healthcare settings, or to workforces beyond healthcare, should follow, rather than precede, evidence from culturally grounded pilots.

## Data Availability

The original contributions presented in the study are included in the article/Supplementary Material, further inquiries can be directed to the corresponding author/s.
