# Supplementary material for: Learning from the world's oldest continuous culture — the circle of leadership: a new way to support the healthcare workforce
Source: Front Health Serv. 2026 Aug 19;6:1888055. doi: 10.3389/frhs.2026.1888055 (PMC13533904; doi:10.3389/frhs.2026.1888055)
Supplement: Supplementary file 1 [file Table1.docx]

**Supplementary Material**

*Supplementary material for the Perspective article: “Learning from the World’s Oldest Continuous Culture — The Circle of Leadership: A New Way to Support the Healthcare Workforce.”*

**Supplementary Figures**

**Supplementary Figure S1. Core Components of Circular Leadership.** Three interconnected components of the Aboriginal-inspired Circular Leadership Model: (1) Structured Mentorship Circles grounded in Aboriginal communal learning, encompassing peer support circles, multi-generational learning, cross-disciplinary connection, and place-based connection; (2) Circular Decision-Making Protocols based on consensus, including circle meetings with equal voice, consensus-building, story-based learning, and new-worker voice integration; and (3) Collective Responsibility for Wellbeing, focused on community wellbeing through shared workload models, collective check-ins, Elder support systems, and community healing practices. The three components feed into and from a shared core of circular leadership in which all components are interconnected and leadership flows through relationships.
